# Versatile Deposition of Complex Colloidal Assemblies from the Evaporation of Hanging Drops

**DOI:** 10.1002/advs.202307893

**Published:** 2023-12-15

**Authors:** Jacopo Vialetto, Théophile Gaichies, Sergii Rudiuk, Mathieu Morel, Damien Baigl

**Affiliations:** ^1^ PASTEUR, Department of Chemistry École Normale Supérieure PSL University Sorbonne Université CNRS Paris 75005 France; ^2^ Department of Chemistry and CSGI University of Florence via della Lastruccia 3, Sesto Fiorentino Firenze I‐50019 Italy

**Keywords:** colloidal crystals, microparticles deposition/patterning, self‐assembly, structural colors, surfactant

## Abstract

Existing strategies designed to produce ordered arrangements of colloidal particles on solid supports are of great interest for their wide range of applications, from colloidal lithography, plasmonic and biomimetic surfaces to tags for anti‐counterfeiting, but they all share various degrees of complexity hampering their facile implementation. Here, a drastically simplified methodology is presented to achieve ordered particle deposition, consisting in adding micromolar amounts of cationic surfactant to a colloidal suspension drop and let it evaporate in an upside‐down configuration. Confinement at the air/water interface enables particle assembly into monolayers, which are then transferred on the substrate producing highly ordered structures displaying vivid, orientation‐dependent structural colors. The method is compatible with many particle types and substrates, while controlling system parameters allows tuning the deposit size and morphology, from monocrystals to polycrystalline disks and “irises”, from single‐component to crystal alloys with Moiré patterns, demonstrating its practicality for a variety of processes.

## Graphical abstract

Simply drying a colloidal suspension drop containing micromolar amounts of surfactant in a hanging configuration enables the deposition of a variety of highly ordered particle arrays displaying vivid structural colors. This includes single crystals and polycrystalline disks (top right), phase‐separated crystal monolayers of binary mixtures (bottom left), crystal alloys forming Moiré patterns (bottom middle), and polycrystalline “irises” (bottom right).

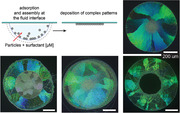

## 1 Introduction

Monolayers of colloidal particles are of widespread interest in the scientific community thanks to their emergent collective properties, both for technological applications^[^ ^1^, ^2^ ^]^ and as macroscopic counterparts of molecular self‐assembly and phase transition phenomena.^[^ ^3^, ^4^ ^]^ Common applications such as particle lithography,^[^ ^5^ ^]^ coatings with controlled wettability,^[^ ^6^ ^]^ plasmonic devices^[^ ^7^ ^]^ or design of biomimetic surfaces,^[^ ^8^ ^]^ seek for periodically‐ordered arrangements of particles that span over millimetric to centimetric areas, making the control of two‐dimensional (2D) crystallization^[^ ^9^ ^]^ one of the main challenge in surface patterning by particles. Over the existing crystallization methods, the guided self‐assembly of colloids is a promising route for the rapid and scalable synthesis of these complex materials. Dispersion of particles can be assembled into ordered monolayers either directly on a solid substrate, or at a liquid interface prior to deposition on the targeted support. Convective horizontal^[^ ^9^, ^10^ ^]^ or vertical assembly^[^ ^11^ ^]^ and spin coating^[^ ^12^, ^13^ ^]^ fall under the first category, while belong to the second one a series of techniques that make use of Langmuir troughs^[^ ^14^, ^15^ ^]^ or particle injection,^[^ ^5^, ^16^ ^]^ among other methodologies,^[^ ^17^, ^18^ ^]^ to first crystallize and then transfer colloidal monolayers. These methods are efficient to obtain precise deposition of large homogeneous monolayers but require complex experimental protocols and are hence often limited to specialized labs.

Alternatively, the simple evaporation of a particle's dispersion confined in a drop has been implemented to directly deposit, or print,^[^ ^19^ ^]^ colloidal assemblies on a substrate. Indeed, both past and recent developments in particle self‐assembly addressed the drying of sessile drops of colloidal suspensions as a direct and simple method for the fabrication of functional materials.^[^ ^20^ ^]^ However, a major limitation for the formation of an ordered monolayer from evaporating drops is the so‐called “coffee‐ring” effect,^[^ ^21^ ^]^ denoting the transport and accumulation of particles toward the pinned drop contact line. As a result, rings^[^ ^21^, ^22^ ^]^ or stripes pattern^[^ ^23^ ^]^ composed of several particle layers are commonly obtained. Such an effect can be controlled or suppressed following various strategies,^[^ ^24^ ^]^ resulting in the formation of domes^[^ ^25^ ^]^ or homogeneous deposits^[^ ^26^, ^27^, ^28^, ^29^ ^]^ with controlled thickness. For example, when drops are deposited on partially wettable surfaces, conditions in which the drop evaporates with a constant contact angle and decreasing contact area minimize the evaporative gradient, leading to the formation of uniform 3D colloidal crystals with spherical cap geometry.^[^ ^30^, ^31^ ^]^ Fewer available strategies have proven suitable for the deposition of ordered monolayers,^[^ ^32^ ^]^ and are often limited to functionalized nanoparticles^[^ ^33^ ^]^ or engineered microgel particles,^[^ ^34^, ^35^ ^]^ to minimize sedimentation and favor adsorption to the air/water (a/w) interface rather than to the substrate. More generic approaches take advantage of controlled evaporation^[^ ^36^ ^]^/condensation,^[^ ^37^ ^]^ often using a cosolvent^[^ ^19^ ^]^ or additives like ethylene glycol or formamide,^[^ ^38^ ^]^ but remain limited to relatively small defect‐free monolayers and require a tightly controlled, and often long, evaporation process.

In this article, we present a novel methodology to deposit on solid substrates monolayers formed by the self‐assembly of colloidal particles, with the idea that this method should be: i) easy to use for non‐specialists and without requiring any equipment or complex chemical additives, and ii) versatile in terms of particle properties (composition and size) that can be self‐assembled. Our strategy consists in depositing drops of aqueous suspension of anionic particles with minute amounts of cationic surfactant on a solid substrate, and then flip them upside‐down to benefit from particles sedimentation and adsorption at the a/w interface, and progressively form a packed monolayer at the interface prior to complete evaporation of the solvent. The evaporation of colloidal suspensions in hanging drops has already been the subject of several studies.^[^ ^39^, ^40^, ^41^ ^]^ However, deposits formed by 2D arrays of ordered particles, to our knowledge, have never been reported, and only transitions from common rings to more flat or dome deposition were evidenced. Interestingly in our system, we demonstrate that control of particles adsorption at the interface, using minute amounts of ionic surfactant,^[^ ^42^, ^43^, ^44^, ^45^ ^]^ and of the wetting properties of the evaporating drops allow for direct deposition of millimetric disk‐like monolayers on the substrate. The deposit size and the crystalline morphology obtained after solvent evaporation were found to be dependent on particle size, relative density, and concentration, as well as on the drop volume; allowing to obtain either a monolayer composed of a single domain of ordered particles, or 2D polycrystalline deposits up to 2 mm in diameter. In addition, we show how the characteristics of this simple method can be advantageously exploited for the fabrication of more complex colloidal structures, not easily accessible by common strategies. We report the formation of mixed co‐assembled patterns of particles separated by size, a simple technique to arrange ordered monolayers one on top of the other, and an original strategy to obtain iris‐like patterns made of polycrystalline rings with particle‐free centers.

## 2 Results and Discussion

### 2.1 2D Colloidal Crystalline Assemblies on Solid Substrates by Drying Hanging Drops

**Figure 1:**
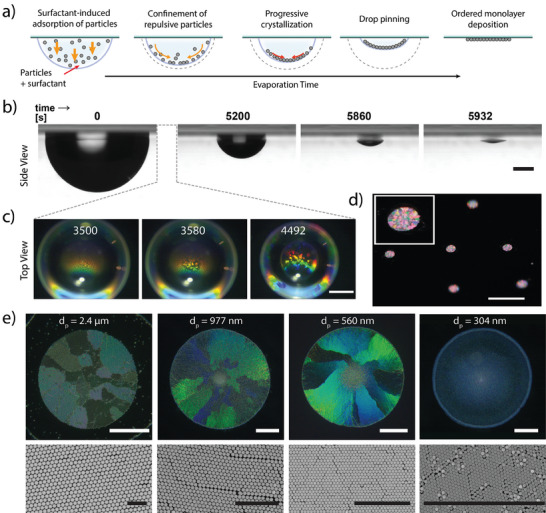
Formation of 2D colloidal crystal structures on a solid substrate from the drying of hanging drops containing anionic silica particles (diameter *d_p_*) at a concentration *C_p_* and minute amounts of DTAB cationic surfactant at a concentration *C_s_*. a) Schematic of the crystallization and deposition process. b) Side‐view transmission microscopy images taken over time during drying of a 5 µL drop (*d*_p_ = 560 nm; *C*_p_ = 0.05 mg mL^−1^; *C*_s_ = 10 µM) deposited on a polystyrene Petri dish and immediately flipped. Scale bar: 500 µm. c) Top‐view optical microscopy images focusing at the air/water interface, taken with the same conditions as in b at different time points. Scale bar: 500 µm. d) Reflection image of five deposits obtained by drying 5 µL drops (*d*_p_ = 2.4 µm; *C*_p_ = 0.05 mg mL^−1^, *C*_s_ = 5 µM) on a glass coverslip. Scale bar: 5 mm. e) Optical microscopy (top) and scanning electron microscopy (SEM) images (bottom) of deposits obtained by drying 5 µL drops containing *C*_s_ = 10 µM and particles of different diameters on a polystyrene Petri dish: *d*_p_ = 2.4 µm (*C_p_* = 0.1 mg mL^−1^), *d*_p_ = 977 nm (*C_p_* = 0.1 mg mL^−1^), *d*_p_ = 560 nm (*C*_p_ = 0.05 mg mL^−1^) and *d*_p_ = 304 nm (*C_p_* = 0.04 mg mL^−1^). Scale bars: 200 µm for microscopy images, 10 µm for SEM images.

**Figure** **1** depicts a typical experiment: a 5 µL aqueous drop containing silica particles (diameter *d*_p_ = 300–2400 nm, concentration *C*_p_ = 0.04–0.1 mg mL^−1^) and minute amounts of dodecyltrimethylammonium bromide surfactant (DTAB, concentration *C_s_* = 5–10 µM) was deposited on a polystyrene (PS) Petri dish, turned upside‐down and left drying. The Petri dish was kept closed to avoid air flows, while temperature and relative humidity were maintained at *T* = 20.5 ± 1 °C and *RH* = 30 ± 5%. Due to their high density, particles were first sedimenting, getting adsorbed at the a/w interface thanks to the surfactant added,^[^ ^42^, ^43^ ^]^ and then accumulated as a monolayer toward the center of the drop (Figure 1a). Meanwhile, the volume of the drop was progressively reduced by evaporation (Figure 1a,b; Movies S1 and S2, Supporting Information). On a PS substrate, the absence of pinning led to an evaporation process at constant receding contact angle *θ*_c_ *=* 84° (Figure S1, Supporting Information), thus progressively reducing the contact drop diameter *D*_drop_ while maintaining a high curvature over the whole interface. In the case of 560 nm diameter particles, we observed after 60 min the appearance of bright and angle‐dependent structural colors at the drop surface (Figure 1c; Movie S3, Supporting Information) indicating the formation of periodic assemblies of particles at the a/w interface. This crystallization process, starting from the center of the drop and propagating toward its edges, is attributed to the 2D confinement of repulsive particles self‐organizing in the curved interface. After 90 min, the interface pinned probably due to the presence of the particle assembly near the contact line. At that time, the contact angle started to decrease at fixed *D*_drop_ until final evaporation and particle deposition (gray dashed line in Figure S1, Supporting Information). The resulting disk‐like deposits kept displaying structural colors on both glass (Figure 1d) and polystyrene (Figure 1e, *top*) indicating successful transfer of ordered particle arrays on the substrate. Scanning electron microscopy (SEM) analyses of the deposits revealed a close‐packed particle assembly with hexagonal order (Figure 1e, *bottom*). This particle organization allowed one to directly visualize the polycrystalline structure of the deposits regardless of particle size for *d_p_* ≥ 560 nm, through the structural color distribution arising from juxtaposing domains with various orientations relative to the light source (Figure 1d,e, *top*). For particles of smaller diameter (*d*_p_ = 304 nm, Figure 1e, *right*), we could still observe the local hexagonal packing by SEM but the structural coloration of the deposit appeared homogenous, probably due to irradiation conditions mismatching the periodic grating. These results show that anionic particles sedimented in the upside‐down drop and adsorbed at the curved a/w interface, where they self‐confined and organized into polycrystalline arrays, a process that could be directly visualized with large particles (*d*_p_ = 2.4 µm, Movie S4, Supporting Information). The particle organization was maintained upon the evaporation‐driven deposition leading to the direct and facile preparation of polycrystalline particle arrays on solid substrates. A key control parameter is the particle settling time, function of the particle diameter, density mismatch with the solvent and fluid viscosity, which has to be shorter than the solvent evaporation time, in turn, dependent on the initial drop volume, temperature, and vapor content, to ensure efficient adsorption at the fluid interface prior to deposition. Interestingly, the resulting solid deposit remained firmly tethered to the substrate, and particles attached to each other by strong attractive surface forces after drying.^[^ ^46^, ^47^ ^]^ This allows for easy manipulation of the self‐assembled disks on their support, either for their direct use or for further implementation in materials of interest. For instance, as proof‐of‐concepts, we successfully adapted conventional microfabrication methods to embed or replicate the colloidal polycrystalline structures into various polymeric materials (photoresist, silicone rubber) while keeping the unique polycrystalline structural coloration signatures (Figure S2, Supporting Information). Used as is or further combined with desired materials, the resulting assemblies could thus be useful in applications such as encryption^[^ ^48^, ^49^ ^]^ or colloidal painting.^[^ ^50^ ^]^

### 2.2 Role of Surfactants and Particle Adsorption at the a/w Interface

We next explored the physico‐chemical determinants of this self‐assembly mechanism. A key‐component in our method is the adsorption of particles at the a/w interface to favor monolayer against multilayer assembly. This step was ensured by the small concentration of cationic surfactant used (*C_s_* = 5–10 µM), which reduced electrostatic repulsion between the negatively charged particles and the a/w interface.^[^ ^42^, ^43^ ^]^ To confirm the need for surfactant, a 5 µL drop containing particles (*d_p_* = 560 nm, *C*_p_ = 0.05 mg mL^−1^) but no surfactant was left drying using the same method. In this case, the anionic particles did not adsorb at the a/w interface due to the electric potential barrier, resulting in a drop diameter continuously decreasing (Movie S5, Supporting Information) and the formation of a thick multi‐layer deposit (Figure S3a, Supporting Information). The deposit showed long‐range ordering due to the progressive packing of highly charged particles but no monolayer was formed. Conversely, in presence of high concentration of DTAB (*C_s_*  =  1 mM), a disordered deposit with particles spread over the whole substrate and a local gel‐like structure was obtained (Figure S3b, Supporting Information). This was due to the adsorption of cationic surfactant on the oppositely charged particle surface strongly decreasing the inter‐particle electrostatic repulsion potential and thus hampering ordered arrangement. Using a low but non‐zero concentration of cationic surfactant, typically of the order of a few µM for DTAB (i.e., ≈10^−3^ CMC where CMC is the critical micelle concentration), was thus necessary to enable the adsorption of the charged particles at the interface and the deposition of an ordered monolayer. Starting from such a low concentration, the effective increase in *C_s_* upon drop drying will reach mM concentration only at a very late stage of evaporation when particles are already assembled and organized at the apex of the drop. We  noticed that a small multi‐layered structure could be seen in the center of some deposits, especially those involving smaller particles (Figure 1e; Figure S4, Supporting Information). We attribute this local multilayer formation to particles tardively reaching the fluid interface where they could not adsorb due to the already formed monolayer, especially at its center where the compression forces from the surrounded adsorbed particles were at maximum. Obtaining perfect monolayers without central accumulation was thus possible but only with sufficiently large particles made of silica (*d_p_* = 2.4 µm) and with even higher diameters for those made of a lower density material such as polystyrene (*d*_p_ = 5.1 µm, Figure S5, Supporting Information). In an additional control experiment, the typical surfactant/particle mixture (*d*_p_ = 560 nm, *C*_p_ = 0.05 mg mL^−1^, *C_s_*  =  10 µM) was dried in upright configuration (Figure S6, Supporting Information), thus removing the gravity‐driven transport of particles and their strong confinement at the a/w interface. Compared to the same experiments done with the hanging drop (Figure 1e, *d*_p_ = 560 nm), the final deposit was composed of a large number of much smaller domains exhibiting structural colors. In that case, particles reaching the descending interface upon evaporation could adsorb, thus forming a monolayer, but the confinement could only be achieved very locally by the collective sinking of particles^[^ ^51^ ^]^ deforming the interface against its curvature and explaining the formation of domains of micrometer size only. Hanging the drop had thus a dual role of both accelerating the transport of particles to the interface and favoring long‐range order by gravity‐driven confinement in the convex fluid interface.

### 2.3 Control of Deposit Morphology by Drop Curvature and Particle Number

**Figure 2:**
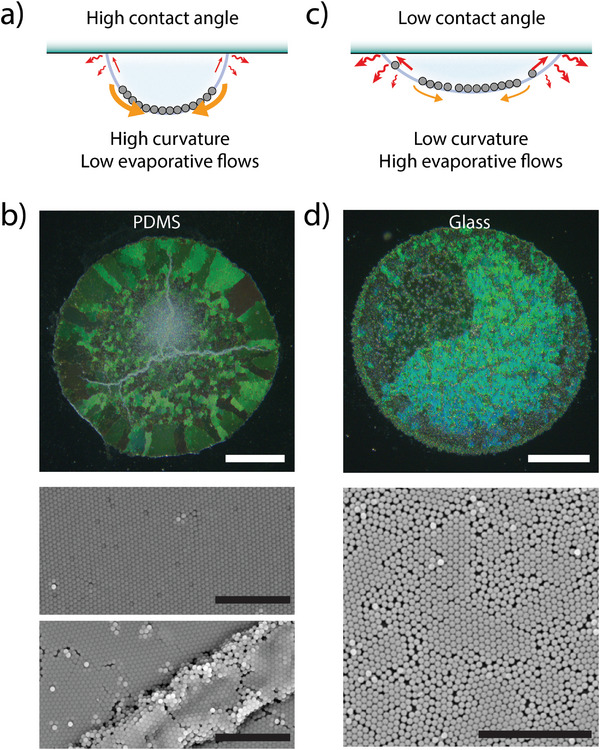
Effect of drop curvature and evaporative flows on the deposit morphology. a) Schematic of the forces acting on the monolayer compression in the case of a high contact angle with the substrate. b) Reflection microscopy (top) and SEM images (bottom) of a deposit obtained from a 5 µL drop of colloidal dispersion on a polydimethylsiloxane (PDMS) surface (silica particles; *d_p_* = 560 nm; *C*_p_ = 0.05 mg mL^−1^; *C*_s_ = 10 µM). c) Schematic of the forces acting on the monolayer packing in the case of a low contact angle with the substrate. d) Reflection microscopy (top) and SEM image (bottom) of a deposit obtained with the same drop composition as in (b) but on a glass coverslip. Scale bars: 200 µm for reflection microscopy images, 10 µm for SEM images.

While adsorption of particles at the fluid interface is necessary to obtain the 2D confinement leading to self‐assembly of an ordered monolayer, the interface geometry itself may play an important role in the final deposit morphology. We were expecting the packing force to increase with particle weight and interface curvature, while evaporation might counteract this phenomenon by creating surface flows toward the drop edges.^[^ ^52^, ^53^ ^]^ Both phenomena were strongly influenced by contact angle and pinning of the contact line, and were explored using different substrates with the typical drop composition (silica particles, *d*_p_ = 560 nm, *C*_p_ = 0.05 mg mL^−1^, *C_s_* = 10 µM). First, using polydimethylsiloxane (PDMS) substrate, a drop with increased contact angle was obtained (*θ*_c_ *=* 106°). In this configuration, the higher curvature was assumed to increase the gravity‐based compressive forces driving the monolayer crystallization, and to reduce the opposing recirculating flows (**Figure** **2**a). Accordingly, the obtained deposit showed numerous long‐range ordered domains (Figure 2b). The relatively small size of domains might be attributed to a faster packing process, which froze boundaries between adjacent crystallites formed at an early stage. The presence of larger domains at the periphery, where compression is lowered and packing more progressive, confirms this hypothesis. Wrinkles propagating throughout the assembly were also observed on the deposit, revealing excessive compressive forces when the diameter of the drop equaled the one of the particles monolayer during the last stage of evaporation (Movie S6, Supporting Information). In contrast, when the drops were deposited on a glass substrate (*θ_c_ =* 52°), the lower interfacial curvature and stronger evaporation flows at the drop edge led to short‐range ordered structures with numerous defects and voids (Figure 2c,d), presumably due to the lower compression forces. However, the weak packing observed on a hydrophilic substrate could be circumvented by using larger particles, thereby increasing compressive forces and the quality of the crystalline deposit (Figure 1d; Figure S7, Supporting Information).

**Figure 3:**
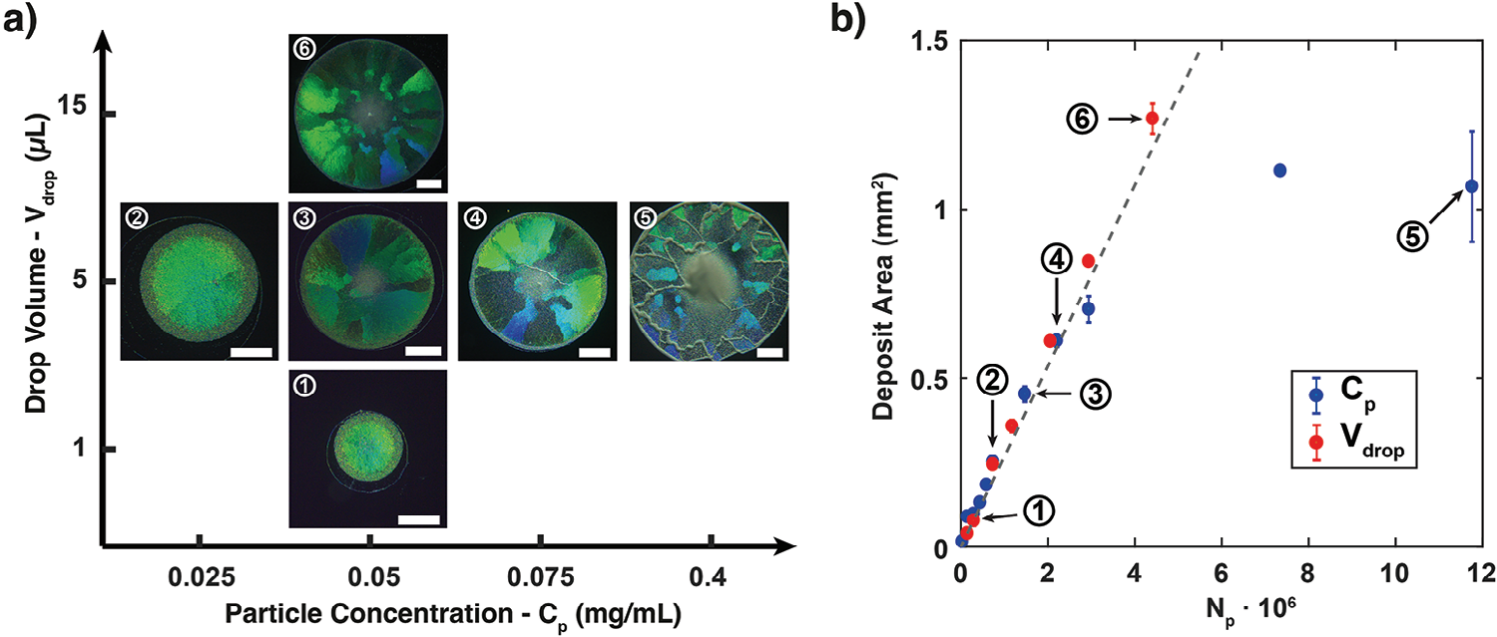
Tuning particle deposits area and morphology as a function of the number of particles (*N_p_*) in the evaporating drops. a) Reflection microscopy images representative of deposits obtained on polystyrene substrate with silica particles (*d*_p_ = 560 nm) at constant *C_s_* = 10 µM and various *C*_p_ and *V*_drop_. The experimental conditions are as follows: 1) *C_p_* = 0.05 mg mL^−1^, *V_drop_* = 1 µL; 2) *C*_p_ = 0.025 mg mL^−1^, *V*_drop_ = 5 µL; 3) *C*_p_ = 0.05 mg mL^−1^, *V*_drop_ = 5 µL; 4) *C_p_* = 0.075 mg mL^−1^, *V_drop_* = 5 µL; 5) *C*_p_ = 0.4 mg mL^−1^, *V*_drop_ = 5 µL; 6) *C*_p_ = 0.05 mg mL^−1^, *V*_drop_ = 15 µL). Scale bars: 200 µm. b) Final deposit area as a function of *N*_p_; *N*_p_ was varied either by changing *C_p_* at fixed *V*_drop_ = 5 µL (blue dots), or by changing *V*_drop_ at constant *C*_p_ = 0.05 mg mL^−1^ (red dots). Representative images in a) are reported in the graph. Dashed line is indicative of the theoretical deposit area for a close‐packed assembly of *N*_p_ particles.

Similar to drop curvature, we hypothesized that increasing particles number (*N*_p_) would affect the structural arrangement in the deposits, either by increasing compaction forces or by adding topological stresses on larger assemblies. To assess the sizes of deposits achievable with our method, and further understand the role of *N*_p_ on the monolayer morphology, we first varied the particles concentration at a fixed drop volume of 5 µL and a constant surfactant concentration *C_s_* = 10 µM (silica particles, *d*_p_ = 560 nm). When increasing *C*_p_ from 0.025 to 0.4 mg mL^−1^ (**Figure** **3**a, conditions 2–5), we observed an expected increase in the disk size (diameter *D*_disk_ ranging from 0.5 to 1.2 mm), but also an increase in the number of monocrystalline domains. This change in deposit morphology was attributed to the collective weight of the particles assembling at the center, hence increasing the local surface deformation,^[^ ^51^ ^]^ and also to geometrical constraints between the sixfold coordinated monolayer and the curved surface so to lower the total energy of the system.^[^ ^54^, ^55^ ^]^ Below a number of particles *N*_p_ = 2∙10^6^, the deposit area increased almost linearly with *N*_p_ and could be compared to the theoretical deposit area for a close‐packed assembly (Figure 3b, blue dots). However, when working at fixed drop volume this relationship was lost for high *N*_p_, leading to smaller deposits than expected with more and more particles depositing on top of the ordered monolayer and wrinkles in the structure (Figure 3a, image 5). This limitation could be circumvented by increasing the drop volume *V*_drop_ at fixed *C*_p_ (Figure 3a, images 1; 3; 6 and Figure 3b, red dots). Larger assemblies could be obtained with the majority of particles in an ordered monolayer, maintaining the linear relationship between particles contained in the drop and the size of the final deposit. Note that a linear relationship was also found between the initial drop area and the final deposit area (Figure S8, Supporting Information). A maximum deposit area of 1.27 mm^2^ was obtained for *V*_drop_ = 15 µL, the higher *V*_drop_ that could be used to form and flip drops that remained held on the substrate. However, the drop volume could be further increased by using a modified technique: first, a 5 µL drop was deposited on the solid substrate and flipped, then an additional volume of the same particle suspension was injected into the flipped drop until the required *V*_drop_ was reached. This allowed to increase almost linearly the final deposit area up to 3.1 mm^2^ for a maximum *V*_drop_ = 55 µL (Figure S8, Supporting Information, gray points), but with the occurrence of wrinkles and multi‐layered regions in the center and at the periphery of the deposit. Conversely, decreasing *N*_p_ (either via *C*_p_ or *V*_drop_) led to the assembly of almost monocrystalline deposits, as characterized by a single structural color in the reflection images (Figure 3a, images 1; 2). All these results emphasize the versatility of this deposition method through the variety of particle type (silica, PS), particle size (from few hundreds of nm to several µm) and substrate nature (PS, PDMS, glass) that can be used, and highlight the degree of control in deposit size and crystalline arrangements achievable through the adjustment of user‐accessible parameters, in particular particle concentration, drop volume and drop curvature through substrate wetting property.

### 2.4 Straightforward Fabrication of Complex Colloidal Assemblies

Next, we explored the flexibility of our method in achieving controlled colloidal deposition into more complex architectures, which can hardly be attained by common deposition strategies.

**Figure 4:**
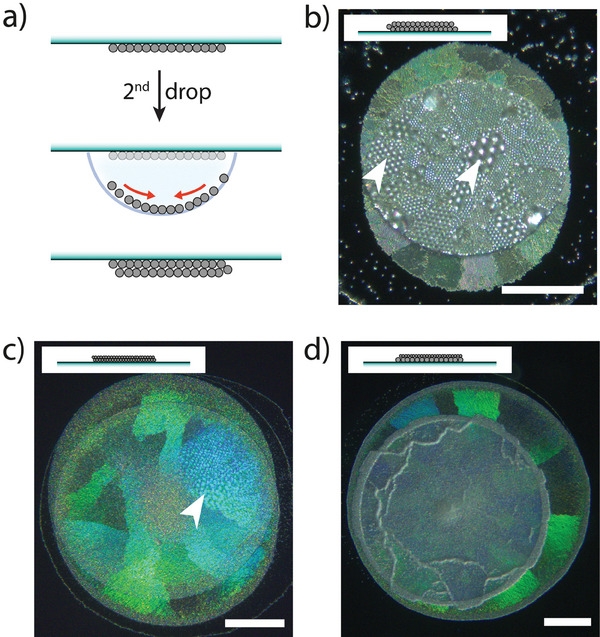
Successive deposition of ordered colloidal monolayers. a) After a first monolayer assembly on a polystyrene substrate, a second drop was deposited on top of the already formed structure, flipped upside‐down and left drying until complete evaporation. b–d) Reflection microscopy of the deposits obtained after drying of the second drop for different composition of particles dispersion (*C*_s_ = 10 µM): b) both drops with *V*_drop_ = 5 µL, *C*_p_ = 0.1 mg mL^−1^ and silica particles *d_p_* = 2.4 µm; c) both drops with *V*_drop_ = 5 µL, *C*_p_ = 0.05 mg mL^−1^ and silica particles *d_p_* = 560 nm; d) first drop: *V_drop_* = 7 µL, *C*_p_ = 0.1 mg mL^−1^ and silica particles *d_p_* = 977 nm, second drop: *V_drop_* = 10 µL, *C*_p_ = 0.05 mg mL^−1^ and silica particles *d*_p_ = 560 nm. Moiré patterns are visible in the reflection images (b) and (c) when the two particle layers have the right relative orientation (white arrows). All scale bars are 200 µm.

First, we took advantage of the adhesion of the dried assemblies on the substrate to repeat the process and add monolayers on top of already formed ones, conveniently producing colloidal materials with structural hierarchies. The successive deposition of ordered particle layers was obtained by using the following experimental procedure: a first monolayer was assembled on the substrate as previously described, then a second drop of particle suspension was carefully placed on top of the already assembled pattern, the substrate was flipped upside‐down and kept in a closed chamber until complete evaporation (**Figure** **4**a). We first deposited either a drop containing 2.4 µm silica particles above an already formed layer of 2.4 µm particles (Figure 4b), or a drop containing 560 nm particles above an already formed layer of 560 nm particles (Figure 4c). After solvent evaporation, a second hexagonal close‐packed monolayer was successfully deposited on top of the first one (SEM in Figure S9, Supporting Information). Interestingly, crystallization at the a/w interface – and not on the previously formed crystalline structure – allowed to keep domains orientation and inter‐particle distances independent between first and second deposition, a characteristic not easily accessible when particles directly assemble on structured substrates.^[^ ^56^ ^]^ The independence of domains orientations between the first and second layer resulted in Moiré interference patterns when the two overlaid monolayers assembled with a small angle displacement (see arrows in Figure 4b,c). We next explored the sequential deposition of particles of different sizes in order to produce layered colloidal crystal “alloys”.^[^ ^57^ ^]^ Deposition of 560 nm particles on 977 nm ones (Figure 4d; Figure S10a, Supporting Information), or 977 nm particles on 2.4 µm ones (Figure S10b, Supporting Information), also led to final crystalline structures in which the assembly of the second layer was independent of the underlying one. The upper layer maintained the same long‐range order both on the bare substrate and on top of the first particle layer (SEM in Figure S10, Supporting Information), where few more defects resulted from the higher roughness of the surface underneath. Overall, this indicates that the monolayers could be deposited also on substrates of complex topologies while keeping their long‐range close‐packed order achieved during the particles self‐organization at the fluid interface.

**Figure 5:**
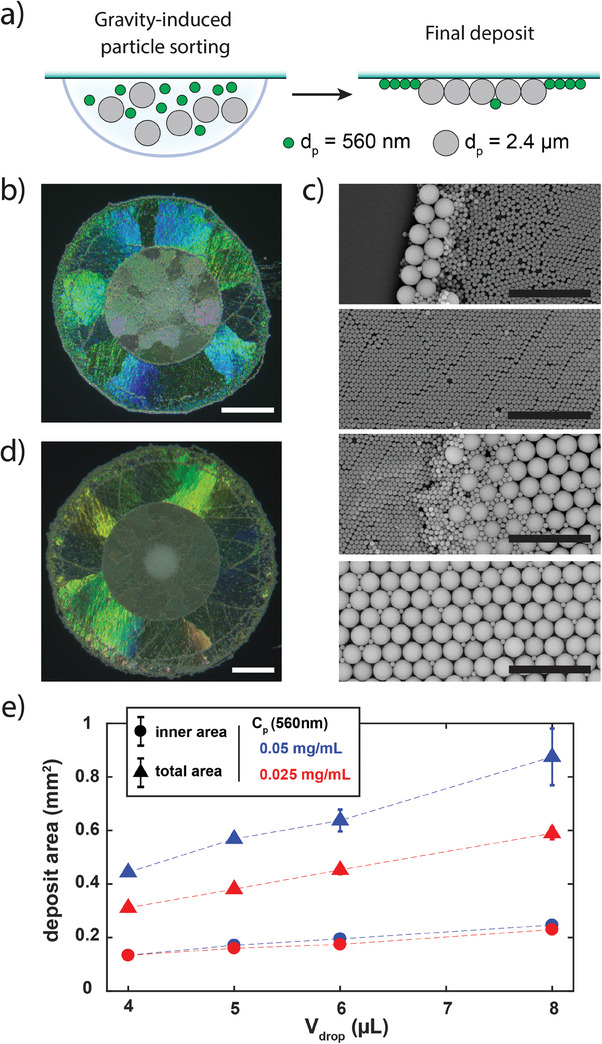
Deposition of phase‐separated colloidal crystal alloys co‐assemblies. a) Schematic of the in‐drop size‐sorting and co‐crystallization of particles with different diameters. b) Reflection microscopy image of a deposit formed on a polystyrene substrate by drying a 5 µL drop containing a mixture of large (*d*_p_ = 2.4 µm, *C*_p_ = 0.1 mg mL^−1^) and small (*d*_p_ = 560 nm, *C*_p_ = 0.05 mg mL^−1^) silica particles, *C_s_* = 10 µM. Scale bar: 200 µm. c) SEM images representative of the co‐crystallization in the deposit obtained in b), showing boundaries and crystalline domains from outer (top) to central region (bottom). Scale bars: 10 µm. d) Reflection microscopy image of a deposit formed on a polystyrene substrate by drying a 8 µL drop with the same composition as in b). Scale bar: 200 µm. e) Areas of the inner deposit (large diameter particles–circles) and total deposit (all particles–triangles) as a function of drop volume *V_drop_*. The concentration of small particles is varied from a 1:2 (red) to a 1:4 (blue) mass ratio to large particles at a fixed *C_p_* = 0.1 mg mL^−1^.

As a second complex hierarchical structure, we investigated the formation of colloidal “alloys” obtained in a single step. We took advantage of the gravity‐dependent accumulation of particles to the drop center to partition particles of different sizes dispersed in the same drop, co‐crystallize them, and deposit the resulting structure on the solid substrate (**Figure** **5**a). Figure 5b shows a typical deposit obtained drying a drop containing a mixture of 2.4 µm and 560 nm silica particles (*V*_drop_ = 5 µL, *C*_s_ = 10 µM, *C*_p_ = 0.1 mg mL^−1^ and *C*_p_ = 0.05 mg mL^−1^ for 2.4 µm and 560 nm particles, respectively). The particles with the bigger diameter sedimented and slid down the curved interface faster than the smaller ones, and gathered at the center of the assembly where they crystallized (Figure 5c, bottom). Only few small particles were trapped in the interstices of the close‐packed hexagonal lattice, without perturbing the long‐range order. Instead, the smaller particles accumulated at the periphery of the central crystal and further crystallized in the outer region (Figure 5c, middle). The transition between the two lattices was characterized by a disordered accumulation of the smaller particles and a higher density of the interstitial particles in the larger lattice. Interestingly, a band of large particles was also observed at the outer rim of the deposit (Figure 5c, top), opposite to what is obtained when drying mixtures of particles from drops in sessile configuration.^[^ ^22^, ^58^ ^]^ This peculiar size‐sorting might be due to the capillary trapping of few particles at the contact line which were eventually merged to the assembly at the last stage of evaporation. By increasing the drop volume (Figure 5d; Figure S11, Supporting Information), or by varying the concentration ratio in the mixed dispersion (Figure 5e), phase‐separated colloidal alloys of different sizes were obtained in a well‐controlled manner. For example, a decrease in the concentration of small particles (small‐to‐big mass ratio of ¼ instead of ½) led to a specific decrease of the outer crystal area, keeping the size of the inner crystal unmodified (red marks in Figure 5e). This demonstrates how ordered assemblies of particles with different diameters could be conveniently obtained in a direct manner exploiting gravity‐controlled size‐sorting at a fluid interface.

**Figure 6:**
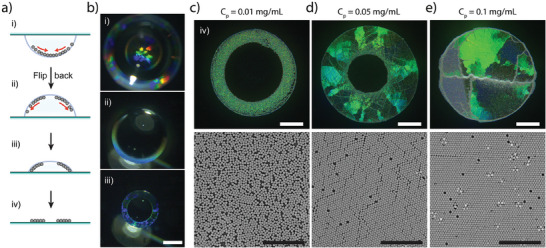
Fabrication of polycrystalline colloidal “irises”. a) Schematic of the “double flipping” method. After initial packing at the center of the hanging drop, the substrate is flipped back and particles trapped at the interface re‐crystallized from the contact line. b) Reflection microscopy images taken during the drop drying process, corresponding to steps i‐iii in a). Scale bar: 400 µm. c–e) Reflection microscopy and SEM images of deposits obtained by using the “double flipping” technique with 560 nm‐diameter silica particles at different *C_p_* (*V_drop_* = 5 µL, *C_s_* = 10 µM) on polystyrene substrate. Scale bars: 200 µm for reflection microscopy images, 10 µm for SEM images.

Last, benefiting from the strong adsorption of particles at the liquid interface, we adapted our deposition method to obtain 2D colloidal “irises”, that is, rings of crystalline monolayer with a particle‐free center. We first deposited on the substrate a drop of the colloidal dispersion, flipped it upside‐down to let particles adsorb at the a/w interface and accumulate at the center of the drop for ≈1 h. The drop was then flipped again and left drying in sessile configuration for an additional hour (**Figure** **6**a). When the drop was upside‐down, particles assembled into a disk‐like 2D crystal, but after flipping to sessile configuration, the particles remained trapped at the a/w interface and rapidly slid down toward the rim of the drop (Figure 6b; Movie S7, Supporting Information). Pre‐assembled large crystalline domains were redispersed into smaller cluster by convective surface flows and progressively recrystallized during the final stage of evaporation (Movie S7, Supporting Information). At fixed drop volume (*V*_drop_ = 5 µL), the final deposit was dependent on the particle concentration, resulting in the formation of different structures as a function of *N*_p_ (Figure 6c–e). When the particle concentration was too low (*C*_p_ = 0.01 mg mL^−1^), the few particles at the interface were not dense enough to collectively crystallize prior to their deposition, therefore forming a ring‐shaped deposit but with no long‐range order and only small hexagonally‐packed clusters (Figure 6c). In contrast, a well‐organized polycrystalline iris was obtained for intermediate particle concentration (*C*_p_ = 0.05 mg mL^−1^, Figure 6d). In that case, particles were in sufficient amount to crystallize from the drop rim after the second flipping, but could not cover the entire a/w interface, therefore splitting into a ring and leaving an empty central region when depositing on the substrate. Finally, for a larger particle concentration (*C_p_* = 0.1 mg mL^−1^), a disk‐like assembly could be formed at the drop a/w interface both before and after the second flipping and it was thus deposited as a whole layer (Figure 6e), in a way similar to the one obtained by drying drops in hanging configuration. Exploiting its simple format, the hanging drop deposition of ordered colloidal monolayer was thus shown to be easily evolvable to more elaborate deposition protocols allowing the controlled deposition of complex colloidal assemblies, ranging from orientation‐independent monolayers assembly to crystal alloys and polycrystalline irises.

## 3 Conclusion

Colloidal monolayers are included in an increasing number of technological applications, from biomimetic and functional coatings to photonics and sensing devices. With the idea of patterning ordered monolayers on a substrate in a direct and straightforward manner, we developed a novel methodology that allows the 2D self‐organization of colloidal particles at the air/water (a/w) interface of a hanging drop and their direct deposition on the targeted substrate upon drop evaporation. The method offers several advantages, including simplicity, versatility in terms of particles and substrates properties, and the ability to obtain ordered monolayers as well as polycrystals of complex structures. The size and crystalline morphology of the deposits was shown to depend on various factors such as particle size, density, concentration, and drop volume. Different strategies, including tuning particles/surfactant concentration or control of wetting properties, were employed to achieve the desired deposition outcomes. Through the exploration of the physico‐chemical determinants of the self‐assembly mechanism, we found that the adsorption of repulsive particles at the a/w interface was crucial for the formation of ordered monolayers, and this was made possible by addition of micromolar concentrations of a conventional cationic surfactant to anionic particles. Following a similar mechanism, we hypothesize that the method might also be applicable without surfactants to pattern particles that spontaneously adsorb at the fluid interface (such as cationic or amphiphilic charged colloids). Gravity‐driven sedimentation of particles was also shown to play a role in the rapid formation of monolayers. Demonstrated with silica particles of different diameters (from 300 nm to 2.4 µm) and larger anionic polystyrene colloids (diameter 5.1 µm), the method thus appears to be readily applicable to any kind of particles capable to sediment at a shorter time scale than the solvent evaporation. The deposit morphology was also influenced by drop curvature and particle number. Particularly, the use of low‐wetting substrates with high drop curvature enhanced the packing forces and led to the formation of highly ordered structures composed of small crystallites, while pinning of the contact line on certain substrates results in lower density of the deposit and larger domains with local defects. Demonstrated on three kinds of surfaces, polystyrene, PDMS, and glass, the established role of the substrate not only allows one to envision enhanced applicability on other substrates (e.g., metals, biomaterials) but also provides guidelines for tuning substrate properties favoring a desired deposit morphology. Additionally, we showed the possibility to easily adapt this “low‐tech” method for the controlled fabrication of complex patterns such as: i) phase‐separated crystal monolayers of binary mixtures, ii) multi‐layered crystals with independently ordered layers thus forming Moiré patterns, or iii) exotic structures such as iris‐like patterns composed of polycrystalline rings displaying vivid structural colors. Consequently, we envision that a number of possible applications can be targeted by this method, exploiting any aspects of its versatility, simplicity, tunability and/or adaptability. This ranges from low‐cost surface patterning and biomimetic materials self‐assembly to photonic devices and other advanced functional materials fabrication. For example, polycrystalline colloidal deposits can be used for anti‐counterfeiting labels due to the formation of unique patterns, providing an easy readout through structural colors and unclonability.^[^ ^48^ ^]^ To conclude, these results represent a simple yet impactful solution for the assembly of functional 2D materials, combining predictable physico‐chemical interactions with a trivial deposition method, in line with the development of novel, yet simpler, routes to advance in materials science.^[^ ^59^ ^]^

## Conflict of Interest

A patent application on the substrate‐tethered polycrystalline colloidal monolayer disks is currently pending to the European Patent Office (Assembly comprising at least one circular polycrystalline colloidal monolayer tethered on a solid substrate. Filed European patent application n° EP22306729.9). The authors declare no other competing interests.

## Supporting information

Supporting Information

Supplemental Movie 1

Supplemental Movie 2

Supplemental Movie 3

Supplemental Movie 4

Supplemental Movie 5

Supplemental Movie 6

Supplemental Movie 7

## Data Availability

The data that support the findings of this study are available from the corresponding author upon reasonable request.
