# Supplementary material for: Versatile Deposition of Complex Colloidal Assemblies from the Evaporation of Hanging Drops
Source: Adv Sci (Weinh). 2023 Dec 15;11(7):2307893. doi: 10.1002/advs.202307893 (PMC10870021; doi:10.1002/advs.202307893)
Supplement: Supplementary file 1 — Supporting Information [file ADVS-11-2307893-s008.pdf]

ADVANCED SCIENCE
Open Access

Supporting Information

for *Adv. Sci.*, DOI 10.1002/advs.202307893

Versatile Deposition of Complex Colloidal Assemblies from the Evaporation of Hanging Drops

*Jacopo Vialetto, Théophile Gaichies, Sergii Rudiuk, Mathieu Morel and Damien Baigl**

Supporting information for:

Versatile deposition of complex colloidal assemblies from the evaporation of hanging drops

Jacopo Vialetto,[1,2] Théophile Gaichies,[1] Sergii Rudiuk,[1] Mathieu Morel[1] and Damien Baigl[1,*]

[1]*PASTEUR, Department of Chemistry, École Normale Supérieure, PSL University, Sorbonne Université, CNRS, 75005 Paris, France.*

[2]*Department of Chemistry and CSGI, University of Florence, via della Lastruccia 3, Sesto Fiorentino, I-50019 Firenze, Italy.*

*E-mail: damien.baigl@ens.psl.eu (DB).

Table of contents

1) Materials and methods

2) Supplementary figures S1–S11

3) Legends of the movies S1–S7

1) Materials and methods

Materials. Ultrapure water (resistivity 18.2 MΩ·cm) was used for all the experiments. The suspensions of negatively charged silica particles, bearing silanol groups on their surface, were purchased from microParticles GmbH. They all had a density ρ = 1.85 g/cm^3 according to the manufacturer. The suspensions of negatively charged polystyrene particles (diameter: 5.1 µm) bearing sulphate and a high density of carboxyl functional groups on their surface were purchased from Life Technologies (CML latex, catalogue number C37255). Although all suspensions were designated as pure, we followed a washing procedure that was found to be essential for obtaining reproducible results. A suspension of the particles at C_p = 10 mg/mL underwent typically six centrifugation cycles where the supernatant liquid was exchanged with ultrapure water each time. Prior to each experiment, each particle suspension was mixed by vortexing (2 min), sonicated in an ultrasound bath (2 min) and vortexed again for another min. Dodecyltrimethylammonium bromide (DTAB, purity ≥ 98%, Sigma-Aldrich) and 1H,1H,2H,2H-Perfluorooctyl-trichlorosilane (purity ≥ 97%, Sigma-Aldrich) were used as received. PDMS was composed of RTV 615 A and RTV 615 B components (Momentive Performance Materials Inc.) and was prepared in ratio 10:1 of monomer/cross-linker. NOA 81 (Norland Optical Adhesive 81, Norland Products) was used as received.

Samples preparation. The proper amounts of water, concentrated particle suspension and concentrated surfactant solution were added in this order in an Eppendorf tube. Then, a drop of the designed volume was deposited on the targeted substrate, flipped upside-down and closed inside a Petri dish to allow drying in a controlled environment, avoiding air flows and possible sample contamination. Double layer particle structures were obtained by carefully depositing a drop of the chosen particle diameter and concentration on top of an already assembled particle layer on the solid substrate. For the "double flipping" technique, a drop of the designed volume was deposited on the targeted substrate, flipped upside-down and closed inside a Petri dish for approx. 1 h in order to accumulate particles to the air/water interface by gravity, then flipped again, this time with the air/water interface up, and let until complete evaporation.

Image acquisition. Side-view microscope images and contact angle measurements were performed with a Drop Shape Analysis System (DSA30, Krüss), by fitting the shape of the drops with the tangent computation method. All top-view microscope images were acquired using a home-built upright microscope, which could operate both in transmission and reflection mode. The microscope mainly consisted of a 12X Zoom Lens System (Navitar), the appropriate microscope objective (20X, NA = 0.42, or 5X, NA = 0.14, Mitutoyo) and an XYZ translation stage on which the samples were placed. White light illumination was provided by a flexible light guide coupled to a cold light source (KL 2500 LED, Schott). The incident white light was either coming with a small angle relative to the air/water interface to obtain structural coloration by reflection, or coming from the bottom of the sample cell, after passing through a diffuser and a mirror, for transmission imaging. A color CCD camera (acA1600-20uc, Basler AG) was used for image acquisition. Dynamics of crystal assembly (Movie S4) was obtained by brightfield transmission microscopy on an inverted microscope (Observer Z1, Zeiss) equipped with a 10X objective and a sCMOS camera (Zyla 4.2 PLUS, Andor Technology).

Scanning electron microscopy images were obtained using a tabletop microscope (Hitachi, TM3000) in *EDX* mode, at magnifications ranging from 180X to 10.000X. The incident angle used was 90° or 45°. Prior to imaging the samples were sputtered with gold (50 mA for 60 s).

Casting of colloidal disks in polymer. The particles were assembled as previously described from drying drops on a polystyrene substrate. A drop of liquid NOA 81 pre-polymer was spread on a glass slide and placed in contact with the already assembled polycrystalline deposits on their substrate. The assembly was irradiated with UV light for 30 s (wavelength: 365 nm, intensity: 10 $mW \cdot cm^{-2}$), and the glass slide with its attached NOA layer was carefully peeled off from the Petri dish. The polymer layer was additionally cured for 2 min under UV light. Alternatively, PDMS liquid pre-polymer (10:1 ratio of monomer/crosslinker) was poured on the already assembled particle crystals on the Petri dish substrate, then the sample was degassed under vacuum to remove bubbles and cured for 3 h at 72 °C. Finally, the formed PDMS slab was carefully peeled off from the Petri dish.

Replica-moulding in polymer. The particles were assembled as previously described from drying drops on a polystyrene substrate. The surface was then activated for 1 min in a plasma oven (Harrick) and silanized overnight in vapour phase using 1H,1H,2H,2H-Perfluorooctyl-trichlorosilane. Liquid pre-polymer (PDMS in 10:1 ratio of monomer/crosslinker) was then poured on it, degassed under vacuum and cured for 3 h at 72 °C. The solidified PDMS was then carefully peeled off, leaving the colloidal assembly tethered on its original substrate. The resulting PDMS mould was then used to replicate the structure in a UV-curable polymer. A drop of liquid NOA 81 pre-polymer was spread on a glass slide and placed in contact with the PDMS mould. The assembly was irradiated with UV light for 2 min (wavelength: 365 nm, intensity: 10 $mW \cdot cm^{-2}$), and the mould was peeled off, leaving a glass slide coated by a patterned NOA layer.

2) Supplementary figures

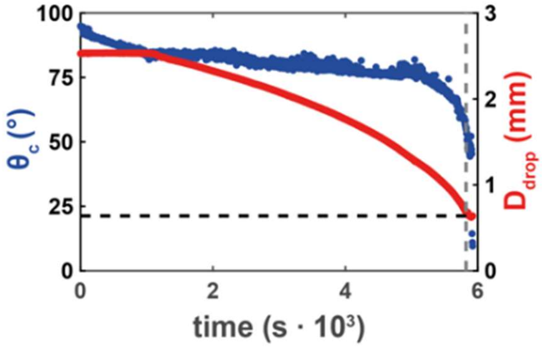

Figure S1. Evolution of contact angle (θ_c) and drop diameter (D_{drop}) as a function of evaporation time for a drying drop as in Fig.1b. The horizontal dashed line indicates the final deposit diameter, the vertical grey dashed line the last pinning of the drop, when particle deposition occurs.

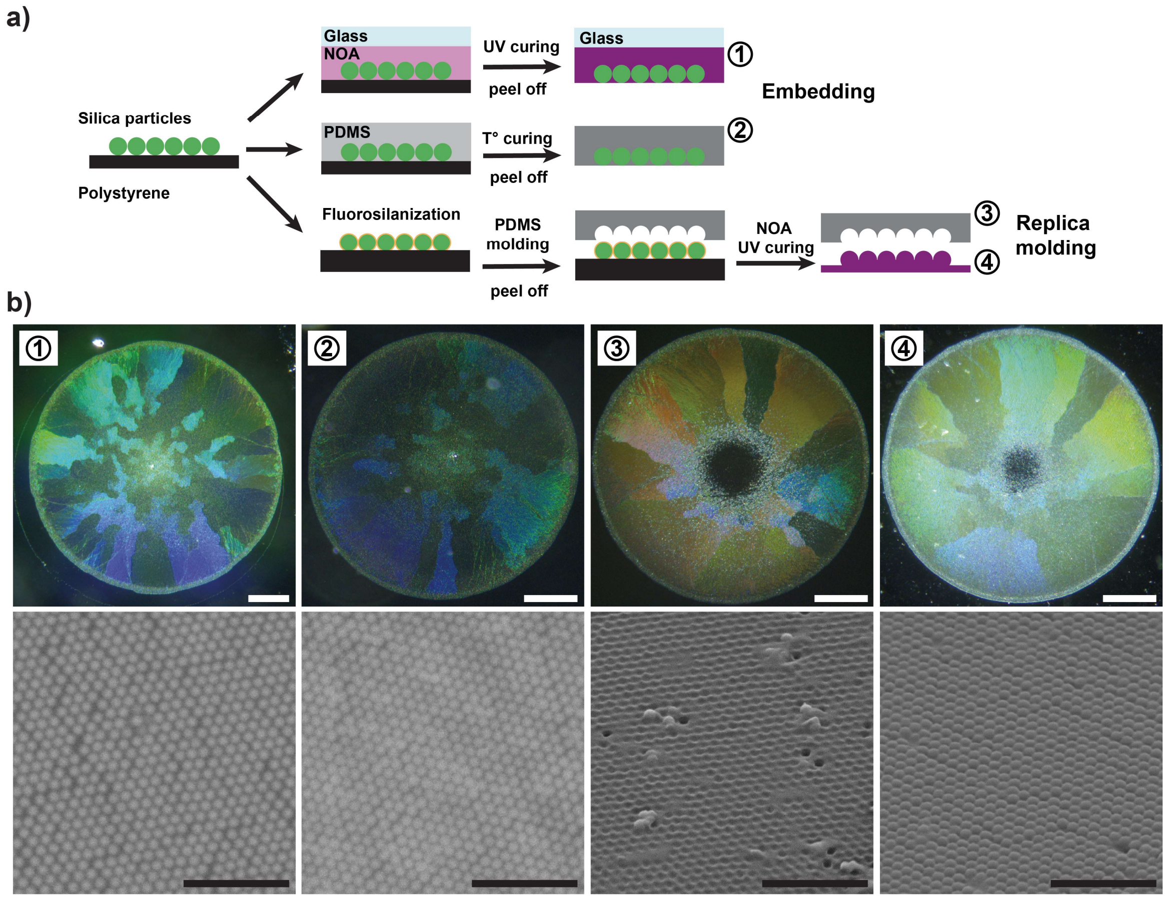

Figure S2. Embedding and replica moulding of deposited monolayers in polymeric materials. **a)** Schematics of the different fabrication methods used for embedding or moulding silica particles monolayer deposited on a polystyrene substrate (see **Materials and methods** for detailed protocols). **b)** Reflection and SEM microscopy images of monolayer transfers described in **a)**: **(1)** particles embedded in NOA 81 UV-curable resist; **(2)** particles embedded in a PDMS slab; **(3)** PDMS mould obtained after surface passivation of particles; and **(4)** NOA 81 replica of the PDMS surface in **(3)**. Scale bars: 200 µm for reflection microscopy images, 5 µm for SEM images.

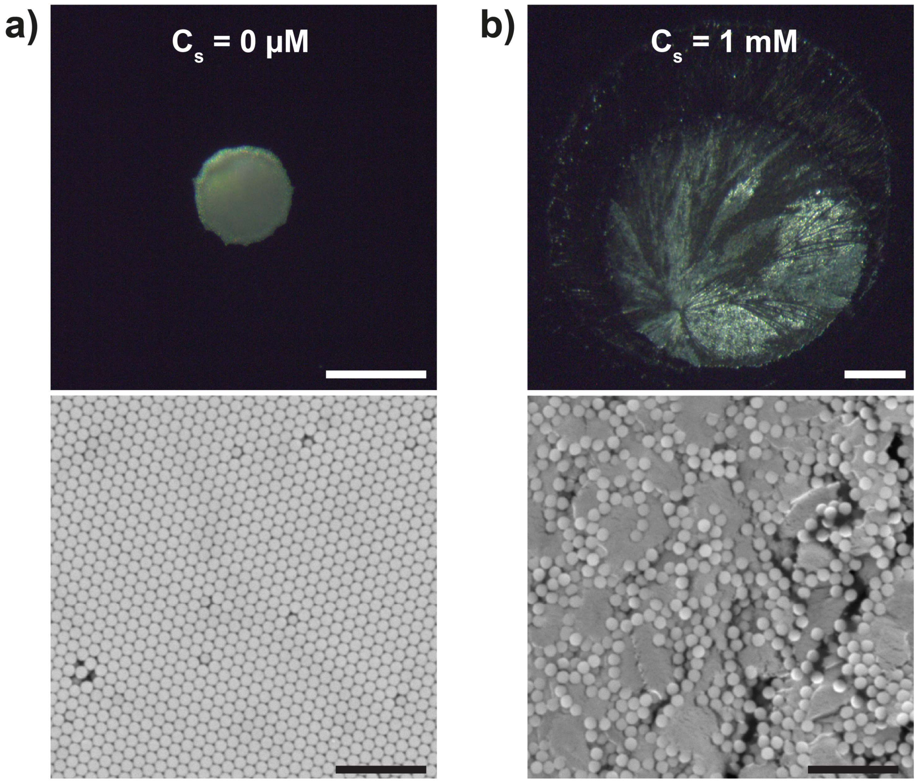

Figure S3. Effect of low and high surfactant concentrations (C_s) on the final particle deposition. **a)** Reflection microscopy and SEM images of a deposit obtained drying a 5 µL drop containing C_p = 0.05 mg/mL and C_s = 0 µM. **b)** Reflection microscopy and SEM images of a deposit obtained after drying a 5 µL drop containing C_p = 0.05 mg/mL and C_s = 1 mM. Cracks in the SEM image are formed during sample imaging. Scale bars: 200 µm for reflection microscopy images, 4 µm for SEM images.

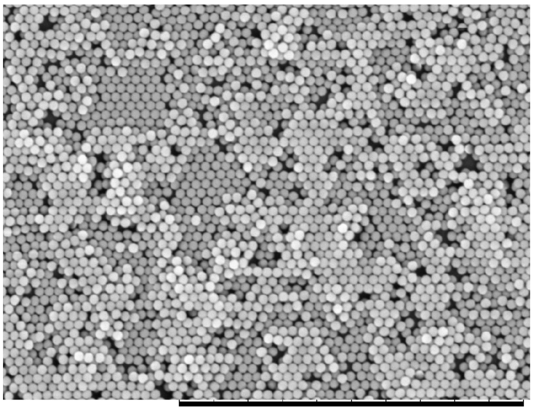

Figure S4. SEM image of the centre of the deposit in Fig. 1e, obtained drying a 5 µl drop containing 560 nm-diameter silica particles (C_p = 0.05 mg/mL, C_s = 10 µM). Scale bar: 20 µm.

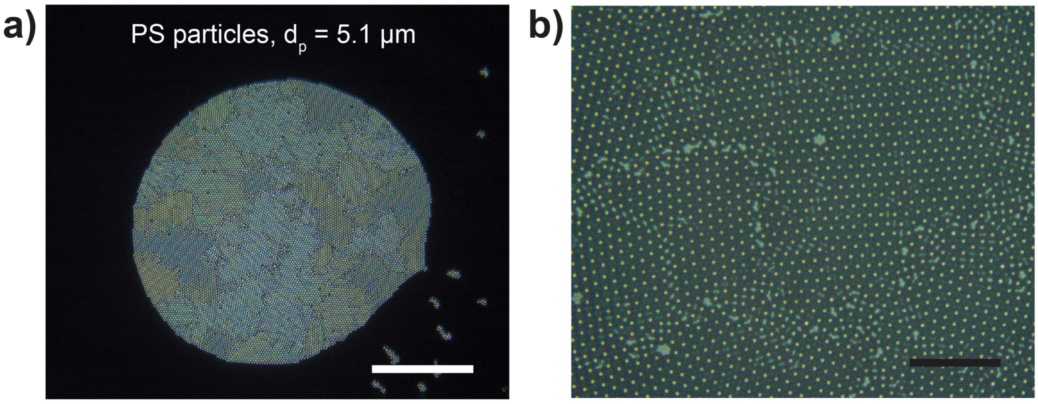

Figure S5. a) Reflection microscopy of the deposit obtained drying a 3 µL drop containing polystyrene particles (d_p = 5.1 µm, C_p = 0.3 mg/mL, C_s = 10 µM). Scale bar: 200 µm. **b)** High magnification transmission microscopy image of the monolayer shown in a). Scale bar: 40 µm.

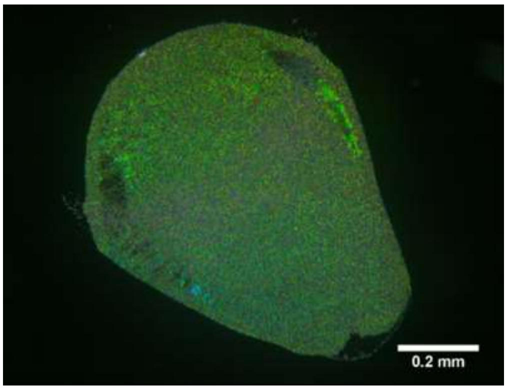

Figure S6. Reflection microscopy image of a deposit obtained drying a 5 µL drop containing the typical composition (C_p = 0.05 mg/mL and C_s = 10 µM) in sessile configuration (upright drying).

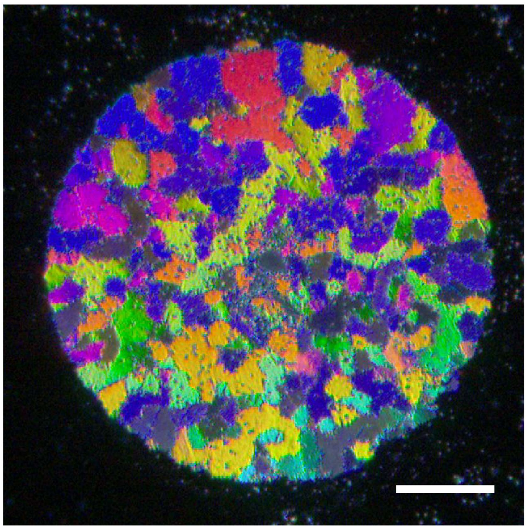

Figure S7. Reflection microscopy image of a deposit obtained drying a 5 μL drop containing large silica particles on a glass substrate (d_p = 2.4 μm, C_p = 0.05 mg/mL, C_s = 5 μM). Scale bar: 200 μm.

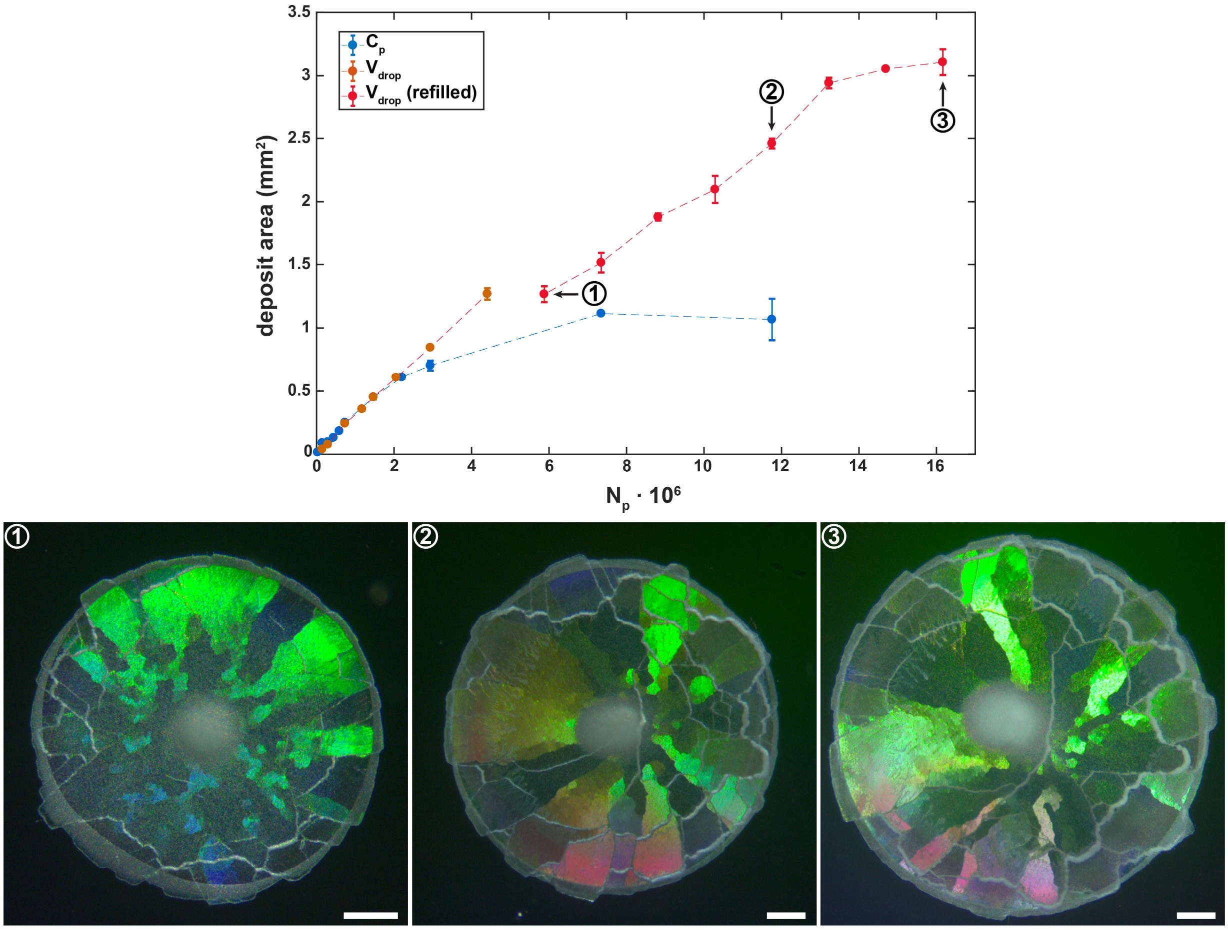

Figure S8. Particle deposits area and their morphology as a function of the number of particles (N_p) in the evaporating drops. Top: Final deposit area as a function of N_p (C_s = 10 µM). N_p was varied either changing C_p at fixed V_{drop} = 5 µL (blue curve), changing V_{drop} at fixed C_p = 0.05 mg/mL (red curve), or increasing V_{drop} by injecting additional volume at fixed C_p = 0.05 mg/mL on a flipped drop (grey curve). Blue and red curves are also displayed in Fig. 3b. Bottom: reflection microscopy images of deposits obtained at increasing V_{drop} (20, 40 and 55 µL for 1, 2 and 3 respectively). Scale bars: 200 µm.

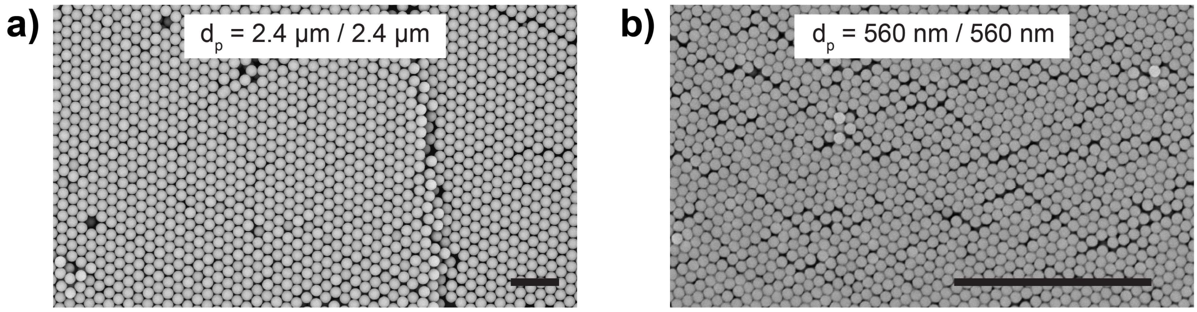

Figure S9. SEM images of the top layer after successive monolayer depositions. **a)** Both drops with V_{drop} = 5 µL, C_s = 10 µM, C_p = 0.1 mg/mL and silica particles d_p = 2.4 µm (reflection microscopy shown in Fig. 4b). **b)** Both drops with V_{drop} = 5 µL, C_s = 10 µM, C_p = 0.05 mg/mL and silica particles d_p = 560 nm (reflection microscopy shown in Fig. 4c). Scale bars: 10 µm.

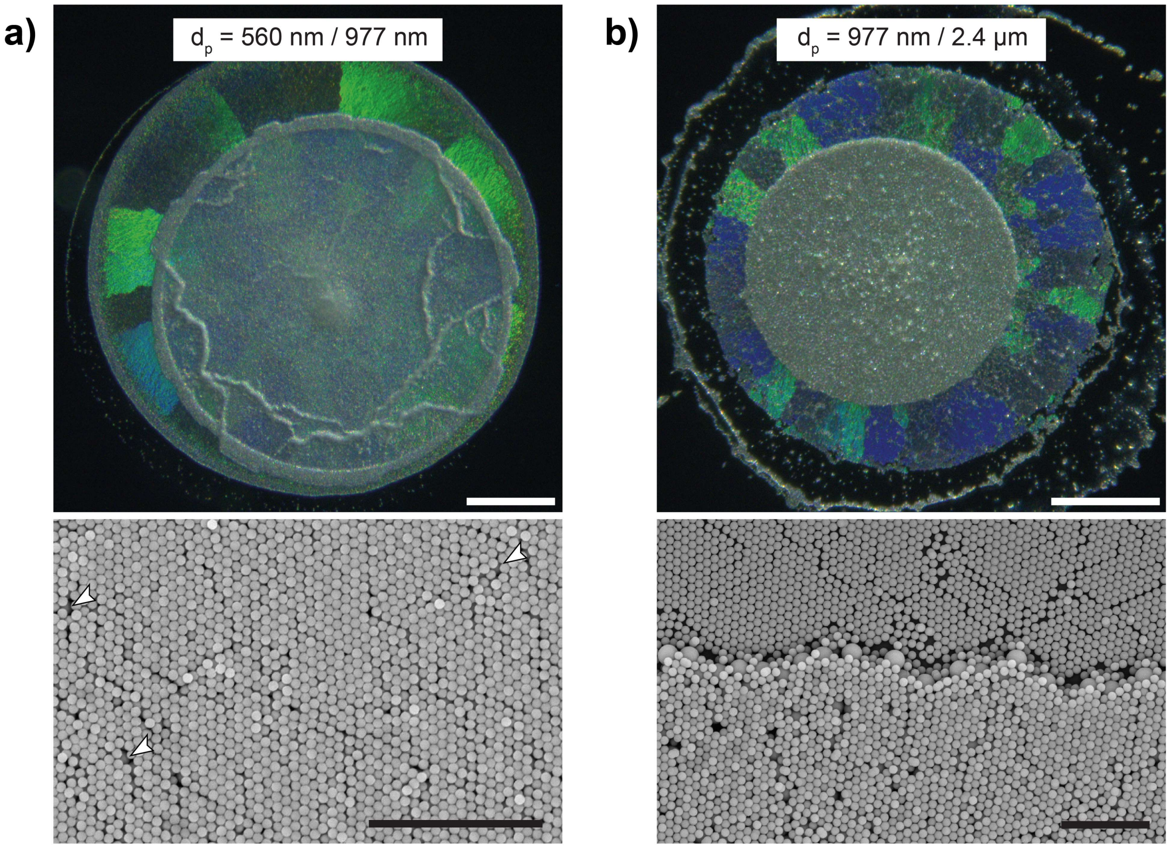

Figure S10. Successive deposition of ordered colloidal monolayers with particles of different diameter. Reflection microscopy and SEM images of the deposits obtained after complete drying of a second drop containing particles of smaller diameter. **a)** Drops composition, first drop: 7 µL, C_s = 10 µM, C_p = 0.1 mg/mL, silica particles d_p = 977 nm; second drop: 10 µL, C_s = 10 µM, C_p = 0.05 mg/mL, silica particles d_p = 560 nm. SEM image is taken in the middle of the top layer, larger particles from the bottom layer can be seen through the lattice defects (arrows). Reflection microscopy image already shown in Fig. 4d. **b)** Drops composition, first drop: 5 µL, C_s = 10 µM, C_p = 0.1 mg/mL, silica particles d_p = 2.4 µm; second drop: 5 µL, C_s = 10 µM, C_p = 0.1 mg/mL, silica particles d_p = 977 nm. SEM image is taken at the border of the first deposition, the crystal lattice of the second deposition is kept on both bottom and top layer. Scale bars: 200 µm for reflection microscopy images, 10 µm for SEM images.

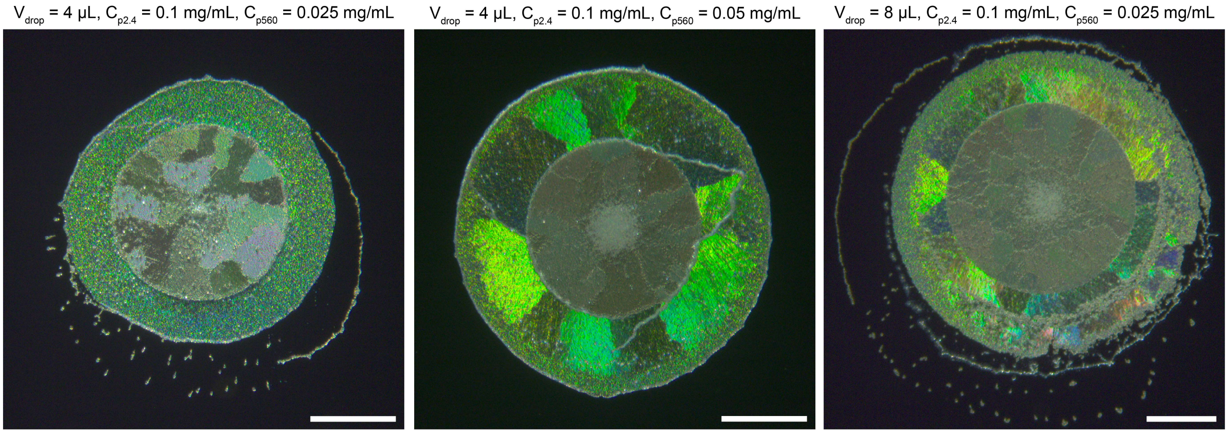

Figure S11. Reflection microscopy images of deposits obtained drying drops containing mixtures of d_p = 2.4 µm and d_p = 560 nm silica particles, at C_s = 10 µM and different V_{drop} and C_p (see Fig. 5e for area of the deposits). Scale bars: 200 µm.

3) Legends of the movies

Movie S1. Drying of a hanging drop, transmission microscopy. Drop composition: 560 nm diameter silica particles, C_p = 0.05 mg/mL, V_{drop} = 5 µL, C_s = 10 µM. The drop was deposited on a polystyrene substrate and turned upside-down before recording the video. The actual time (min:sec) is displayed in the top left corner. Scale bar: 300 µm.

Movie S2. Drying of a hanging drop, side-view transmission microscopy. Drop composition: 560 nm diameter silica particles, C_p = 0.05 mg/mL, V_{drop} = 5 µL, C_s = 10 µM. The drop was deposited on a polystyrene substrate and the video was started 12 minutes after turning the drop upside-down. The actual time (min:sec) is displayed in the top left corner. Scale bar: 300 µm.

Movie S3. Drying of a hanging drop, reflection microscopy. Drop composition: 560 nm diameter silica particles, C_p = 0.05 mg/mL, V_{drop} = 5 µL, C_s = 10 µM. The drop was deposited on a polystyrene substrate and the video was started 5 minutes after turning the drop upside-down. The actual time (min:sec) is displayed in the top left corner. Scale bar: 300 µm.

Movie S4. Crystallization in a hanging drop, transmission microscopy. Drop composition: 2.4 µm diameter silica particles, C_p = 0.05 mg/mL, V_{drop} = 2.5 µL, C_s = 5 µM. The drop was deposited on a polystyrene substrate and the video was started 5 minutes after turning the drop upside-down. Focused frames were manually selected from z-stack acquisition done every 2 minutes in order to follow the air/water interface during the evaporation process. Scale bar: 100 µm.

Movie S5. Drying of a hanging drop in the absence of surfactants in suspension. Drop composition: 560 nm diameter silica particles, C_p = 0.05 mg/mL, V_{drop} = 5 µL. The drop was deposited on a polystyrene substrate and turned upside-down before recording the video in transmission mode. The actual time (min:sec) is displayed in the top left corner. Scale bar: 300 µm.

Movie S6. Drying of a hanging drop on a PDMS substrate. Drop composition: 560 nm diameter silica particles, C_p = 0.05 mg/mL, V_{drop} = 5 µL, C_s = 10 µM. The drop was deposited on a PDMS substrate and the video was started in reflection mode 82 minutes after turning the drop upside-down. Focus was manually adjusted to follow the particle assembly at the air/water interface during the evaporation process. The actual time (min:sec) is displayed in the top left corner. Scale bar: 300 µm.

Movie S7. Fabrication of a polycrystalline colloidal “iris”. Drop composition: 560 nm diameter silica particles, C_p = 0.05 mg/mL, V_{drop} = 5 µL, C_s = 10 µM. The drop was deposited on a polystyrene substrate the video was started in reflection mode approx. 1 hour after turning the drop upside-down. The drop was turned back in sessile configuration 4 minutes after starting the recording. After the second flipping, the focus was manually adjusted to the drop contact line. The actual time (min:sec) is displayed in the top left corner. Scale bar: 300 µm.
